# Closing the mortality gap after a myocardial infarction in people with and without chronic obstructive pulmonary disease

**DOI:** 10.1136/heartjnl-2014-307251

**Published:** 2015-03-12

**Authors:** Kieran J Rothnie, Liam Smeeth, Emily Herrett, Neil Pearce, Harry Hemingway, Jadwiga Wedzicha, Adam Timmis, Jennifer K Quint

**Affiliations:** 1 Faculty of Epidemiology and Population Health, London School of Hygiene and Tropical Medicine, London, UK; 2 Farr Institute of Health Informatics Research, London, UK; 3 Department of Epidemiology and Public Health, University College London, London, UK; 4 National Heart and Lung Institute, Imperial College London, London, UK; 5 Barts NIHR Biomedical Research Unit, Queen Mary University of London, London, UK

## Abstract

**Objective:**

Patients with chronic obstructive pulmonary disease (COPD) have increased mortality following myocardial infarction (MI) compared with patients without COPD. We investigated the extent to which differences in recognition and management after MI could explain the mortality difference.

**Methods:**

300 161 patients with a first MI between 2003 and 2013 were identified in the UK Myocardial Ischaemia National Audit Project database. Logistic regression was used to compare mortality in hospital and at 180 days postdischarge between patients with and without COPD. Variables relating to inhospital factors (delay in diagnosis, use of reperfusion and time to reperfusion/use of angiography) and use of secondary prevention were sequentially added to models.

**Results:**

Mortality was higher for patients with COPD both inhospital (4.6% vs 3.2%) and at 180 days (12.8% vs 7.7%). After adjusting for inhospital factors, the effect of COPD on inhospital mortality after MI was reduced for both ST-elevation myocardial infarctions (STEMIs) and non-STEMIs (STEMIs OR 1.24 (95% CI 1.10 to 1.41) to 1.13 (95% CI 0.99 to 1.29); non-STEMIs OR 1.34 (95% CI 1.24 to 1.45) to 1.16 (95% CI 1.07 to 1.26)). Adjusting for inhospital factors reduced the effect of COPD on mortality after non-STEMI at 180 days (OR 1.56 (95% CI 1.47 to 1.65) to 1.37 (95% CI 1.31 to 1.44)). Adjusting for use of secondary prevention also reduced the effect of COPD on mortality at 180 days for STEMIs and non-STEMIs (STEMIs OR 1.45 (95% CI 1.31 to 1.61) to 1.25 (95% CI 1.11 to 1.41); non-STEMIs OR 1.37 (95% CI 1.31 to 1.44) to 1.26 (95% CI 1.17 to 1.35).

**Conclusions:**

Delayed diagnosis, timing and use of reperfusion of a STEMI, use of angiography after a non-STEMI and use of secondary prevention medicines are all potential explanations for the mortality gap after MI in people with COPD.

## Introduction

People with chronic obstructive pulmonary disease (COPD) are at higher risk of cardiovascular disease^1^ ^2^ and are known to have poorer medium and longer-term outcomes after myocardial infarction (MI) compared with people without COPD, however, findings for inhospital mortality have been mixed.^3–6^ The heterogeneity in findings on inhospital mortality may be due to differences in treatment practices. COPD is currently the third leading cause of death worldwide.^7^ As up to one-third of deaths in people with COPD are due to cardiovascular disease,^8^ reducing deaths after MI in this population is important. In addition, there is a lack of evidence for the effectiveness of treatments in those with comorbidities.

Recent years have seen improvements in outcomes for patients after MI.^9^ However, several recent studies have continued to report poorer mortality for patients with COPD after an MI. Although the reasons for increased mortality after MI in patients with COPD are likely to include biological factors related to COPD, differences in recognition and management between patients with and without COPD may play a role. Recent work has demonstrated that patients with COPD are less likely to receive reperfusion treatment or β blockers after an MI,^10^ and that not prescribing β blockers to patients with COPD impacts on mortality.^11^

Little is known about potential differences in prescribing of other secondary prevention medicines, inhospital treatment or on the effects that any differences in these potentially modifiable factors may have on mortality.

We used Myocardial Ischaemia National Audit Project (MINAP), a national register of hospital care for acute coronary syndromes (ACS), to investigate the extent to which differences in recognition and management of an MI might account for the mortality gap in patients with COPD at the population level.

## Methods

### Data source

The MINAP database is a registry of all admissions for MI and other ACS to hospitals in the UK. The dataset includes information on patient demographics, comorbidities, drugs on admission, initial diagnosis, final diagnosis, inhospital drug treatment, timing of reperfusion therapies, inhospital outcome and drugs given on discharge.^12^

We included all patients with a first diagnosis of ST-elevation myocardial infarction (STEMI) from January 2003 to June 2013 or non-ST-elevation myocardial infarction (non-STEMI) from January 2004 to December 2012. Records were excluded if they did not have a patient unique identifier, if patients had missing values for presence of obstructive airway disease or smoking history or if Office of National Statistics (ONS) mortality data were missing.

### Exposure identification

The obstructive airway disease variable in MINAP does not differentiate between COPD and asthma. In order to identify patients with COPD for this analysis, a strategy was developed and tested in a subset of the data linked with data from the Clinical Practice Research Datalink (CPRD). CPRD is a large UK clinical database of primary care medical records which includes over 5.5 million active patients (8% of the population).^13^ Around half of the CPRD records have been linked to the MINAP database through the CALIBER linkage scheme.^14^ Patients with COPD can be identified in CPRD through the use of validated diagnostic codes. Using this subset of linked data, we developed strategy for identifying COPD patients in MINAP using CPRD-identified COPD as a reference standard. In this subset of data, patients with COPD were identified using a combination of MINAP-recorded obstructive airway disease and a smoking history (ex or current smoker). This strategy resulted in adequate identification of patients with COPD in MINAP, with agreement of 90.9%.

### Outcome definitions

#### Recognition and management

Delay in diagnosis of MI, reperfusion after a STEMI, use of angiography in hospital after a non-STEMI and discharge on secondary prevention drugs were investigated. Two definitions of delay in diagnosis were investigated for patients with a final diagnosis of STEMI: (1) delay in diagnosis of definite STEMI (defined as those who did not have an initial diagnosis of definite STEMI) and (2) delay in diagnosis of ACS (defined as those whose initial diagnosis was not STEMI, probable MI or ACS). For those patients with a final diagnosis of non-STEMI, one definition for delay in diagnosis was investigated: delay in diagnosis of ACS (defined as those whose initial diagnosis was not STEMI, probable MI or ACS).

#### Mortality outcomes

The UK ONS collects data on all recorded deaths in England and Wales. MINAP is linked with ONS mortality data, which provides data on vital status at 180 days postdischarge. Mortality at 180 days postdischarge was assessed for those who survived until discharge.

### Statistical analysis

Demographic and clinical characteristics were tabulated for patients with COPD and non-COPD patients. All analyses were stratified by type of MI (STEMI or non-STEMI). The models were adjusted for smoking status, age, sex and calendar year, comorbidities including prior angina, cerebrovascular disease, chronic kidney failure, diabetes, congestive heart failure, hypertension, hyperlipidaemia, peripheral vascular disease, previous percutaneous coronary intervention (PCI) and previous coronary artery bypass graft and cardiovascular drugs (ACE inhibitor or angiotensin receptor blocker, β blocker, statin and thienopyridine) use on admission. Following the suggested practice for missing data in MINAP,^15^ missing values for comorbidities and drugs on admission were recoded to ‘no’. Other variables were not recoded and analyses were conducted on the basis of complete case analysis. Data were analysed using Stata V.13.0.

Analysis was conducted in three parts:

1. Describing the problem: differences in mortality after MI between patients with COPD and non-COPD patients
  We compared crude proportions of patients with COPD dying inhospital and at 180 days postdischarge to patients without COPD. We then used logistic regression to adjust the comparisons of mortality for possible confounders for age, sex, smoking status, calendar year, comorbidities and drugs used on arrival.
2.  Possible inhospital explanations: differences in recognition and management after an MI between patients with COPD and non-COPD patients
  For STEMIs, we investigated differences in delay in STEMI diagnosis, use of primary PCI (pPCI), use of thrombolysis, time to reperfusion from hospital admission and use of secondary prevention drugs on discharge. We investigated the impact of delay in diagnosis on time to reperfusion, and we assessed whether COPD modified this relationship. For non-STEMIs, we investigated delay in diagnosis of MI, use of angiography in hospital and use of secondary prevention drugs on discharge.
3.  Accounting for differences in mortality after MI between patients with COPD and non-COPD patients in terms of hospital processes
  In order to investigate to what extent differences in diagnosis and treatment of patients with COPD after an MI might account for differences in mortality, variables relating to inhospital processes investigated in (2) were sequentially added to mortality models created in (1) with reference to a directed acyclic graph (see online supplementary material). Attributable risk of death due to COPD following MI was calculated before and after adjustment for inhospital processes using the formula (OR-1)/OR×100.

## Results

### Characteristics of participants

**Figure 1:**
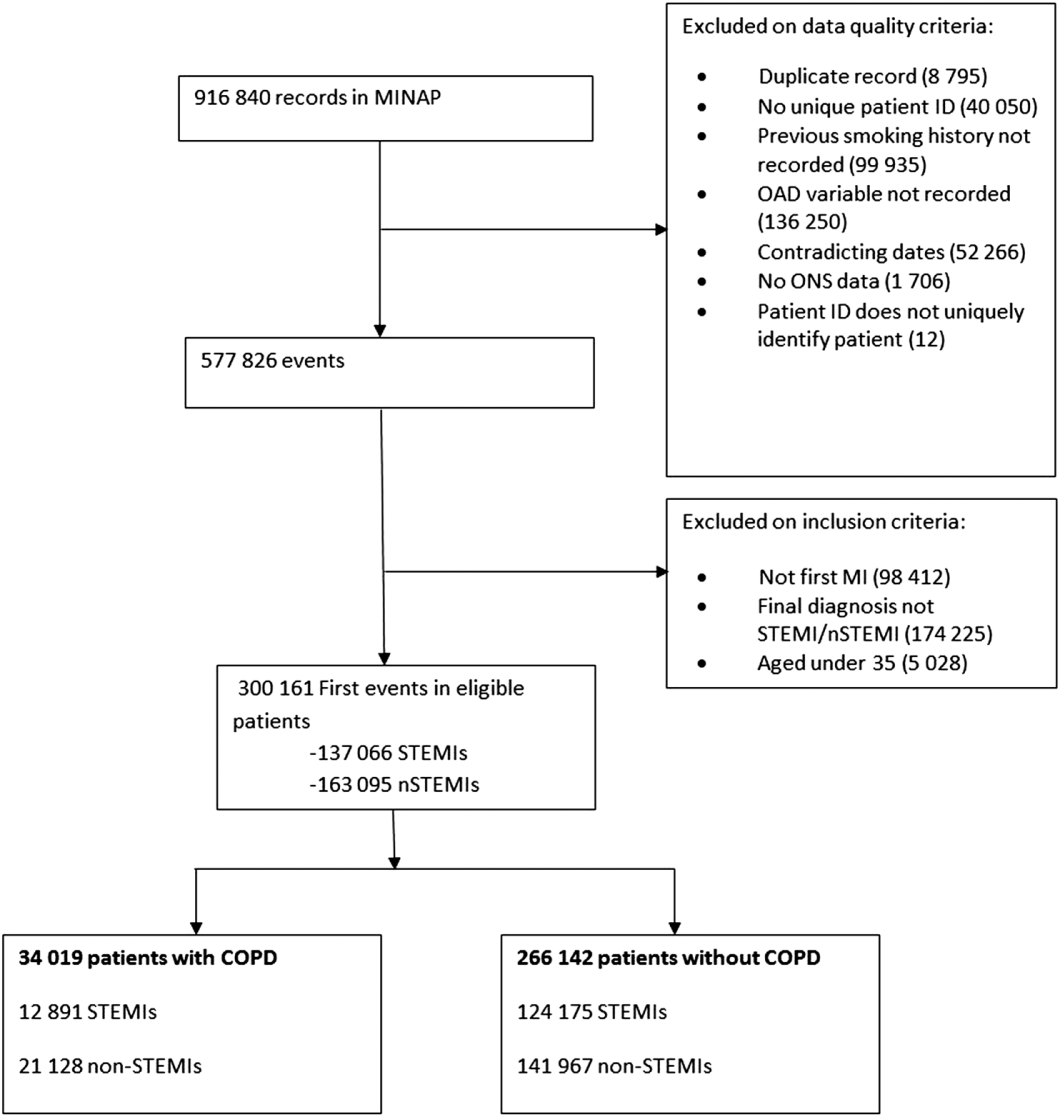
Study selection.

Of the 300 146 patients with first MI identified over the period, 34 027 (11.3%) had COPD. The inclusion and exclusion of records in the MINAP database are detailed in figure 1. The characteristics of the patients included in the study are detailed in table 1. Mortality was higher for patients with COPD both inhospital (4.6% vs 3.2%) and at 180 days (12.8% vs 7.7%).

**Table 1:** Characteristics of patients in the study

Characteristic	COPD n (%)	Non-COPD n (%)
Sex		
Male	21 053 (61.9)	178 611 (67.1)
Female	12 908 (37.9)	86 504 (32.5)
Missing	80 (0.2)	956 (0.36)
Age (years)		
<60	7627 (22.6)	90 557 (34.1)
60–70	8830 (26.0)	62 947 (23.7)
71–80	10 622 (31.3)	61 549 (23.2)
>80	6786 (20.0)	50 126 (18.9)
Missing	0	0
Smoking status		
Current	14 666 (43.2)	90 026 (34.0)
Ex	19 244 (56.8)	87 612 (33.0)
Never	0	87 541 (33.0)
Missing	0	0
Previous Angina		
Yes	7426 (21.8)	41 417 (15.6)
No	25 936 (76.2)	223 089 (83.9)
Missing	679 (2.0)	1565 (0.6)
Previous PCI		
Yes	908 (2.7)	6622 (2.5)
No	32 082 (94.3)	255 449 (96.0)
Missing	1051 (3.1)	3916 (1.5)
Previous CABG		
Yes	786 (2.3)	5704 (2.1)
No	32 227 (94.7)	256 574 (96.4)
Missing	1028 (3.0)	3793 (1.4)
Diabetes		
Yes—diet controlled	1193 (3.5)	8322 (3.1)
Yes—oral	2902 (8.5)	21 418 (8.1)
Yes—insulin	1241 (3.7)	8986 (3.4)
Yes—insulin and oral	176 (0.5)	1178 (0.4)
No	28 030 (82.3)	223 040 (83.8)
Missing	499 (1.5)	3127 (1.2)
Treated for hypertension		
Yes	15 304 (45.0)	117 886 (44.3)
No	18 151 (53.3)	146 459 (55.1)
Missing	586 (1.7)	1726 (0.7)
Treated for hyperlipidaemia		
Yes	9091 (26.7)	73 641 (27.7)
No	23 399 (68.7)	185 043 (69.6)
Missing	1551 (4.6)	7387 (2.8)
Peripheral vascular disease		
Yes	1962 (5.8)	9061 (3.4)
No	30 872 (90.7)	253 720 (95.4)
Missing	1207 (3.6)	3290 (1.2)
Previous cerebrovascular disease		
Yes	2823 (8.3)	16 829 (6.3)
No	30 354 (89.2)	247 418 (93.0)
Missing	864 (2.5)	1824 (0.7)
Heart failure		
Yes	2037 (6.0)	7426 (2.8)
No	31 080 (91.3)	256 677 (96.5)
Missing	924 (2.71)	1968 (0.7)
Renal failure		
Yes	1681 (4.9)	8428 (3.2)
No	31 452 (92.4)	255 732 (96.1)
Missing	908 (2.7)	1911 (0.7)
β blocker on arrival		
Yes	3016 (8.9)	44 585 (16.8)
No	23 544 (69.1)	162 876 (61.2)
Missing	7481 (22.0)	58 610 (22.0)
ACEi/ARB on arrival		
Yes	8228 (24.2)	57 288 (21.53)
No	18 331 (53.9)	150 036 (56.4)
Missing	7482 (22.0)	58 747 (22.1)
Statin on arrival		
Yes	9446 (27.8)	65 062 (24.5)
No	17 409 (51.1)	144 498 (54.3)
Missing	7186 (21.1)	56 511 (21.2)
Thienopyridine on arrival		
Yes	2948 (8.7)	23 240 (8.7)
No	22 729 (66.8)	176 548 (66.4)
Missing	8364 (24.6)	66 283 (24.9)
Death in hospital	1561 (4.6)	8574 (3.2)
Death at 180 days (survivors to discharge)	4166 (12.8)	19 693 (7.7)

ACEi, ACE inhibitor; ARB, angiotensin receptor blocker; CABG, coronary artery bypass graft; COPD, chronic obstructive pulmonary disease; PCI, percutaneous coronary intervention.

### ST-elevation myocardial infarctions

1. Describing the problem: differences in mortality after MI between patients with COPD and non-COPD patients
  After adjusting for age, sex, smoking status, calendar year, comorbidities and drugs on arrival, mortality in patients with COPD was higher than non-COPD patients in hospital (OR 1.24, 95% CI 1.10 to 1.41), and 180 days after discharge (OR 1.45, 95% CI 1.33 to 1.59).
2.  Possible inhospital explanations: differences in recognition and management after an MI between patients with COPD and non-COPD patients
  Differences in diagnosis and inhospital recognition management are presented in table 2. Patients with COPD who had a STEMI were more likely to have an initial diagnosis other than definite STEMI (OR 1.24, 95% CI 1.19 to 1.30) or ACS (OR 1.52, 95% CI 1.42 to 1.62). After a STEMI, patients with COPD were less likely to have pPCI (OR 0.87, 95% CI 0.83 to 0.92). There was no evidence that patients with COPD were less likely to receive thrombolysis (OR 0.96, 95% CI 0.91 to 1.10).
  In adjusted results, differences in time to reperfusion have been expressed in terms of exponentiated linear regression coefficients which, in this case, represent ratios of geometric means. The relationship between COPD and time to reperfusion was found to be different depending on whether diagnosis of MI was delayed (p value for interaction <0.001). The median time to reperfusion was 43.7 min longer for patients with COPD compared with non-COPD patients among those who had a delay in diagnosis (median time to reperfusion 152.9 min (IQR, 74.3–705.6 min) for patients with COPD, and 109.2 min (IQR, 50.2–260.0 min) for non-COPD patients). This difference remained on adjusted analysis and corresponded to 47% (95% CI 15% to 88%) longer time to reperfusion for patients with COPD with delayed diagnosis of MI, compared with non-COPD patients with delayed diagnosis of MI. There was no difference in time to reperfusion between patients with COPD and non-COPD patients among those without a delay in diagnosis (see details in online supplementary appendix). Patients with COPD were less likely to receive any of the secondary prevention drugs, apart from thienopyridines, on discharge compared with non-COPD patients, β blockers significantly more so than other drugs (OR 0.26 (95% CI 0.25 to 0.27)).
3.  Accounting for differences in mortality after MI between patients with COPD and non-COPD patients in terms of hospital processes
  When compared with the result found in (1), inhospital mortality was reduced after adjusting separately for both diagnostic delay (OR 1.20 (95% CI 1.06 to 1.36)) and time to reperfusion and use of pPCI (OR 1.11 (95% CI 0.94 to 1.31; table 3)). After adjusting for all inhospital factors, the OR for mortality was 1.13 (95% CI 0.99 to 1.29). For mortality at 180 days, the OR was 1.45 (95% CI 1.33 to 1.59) after adjusting for age, sex, smoking, calendar year, drugs used on admission and comorbidities, and was 1.45 (95% CI 1.31 to 1.61) after additionally adjusting for diagnostic delay, use of pPCI and time to reperfusion. Adjusting for use of secondary prevention drugs on discharge substantially reduced ORs for 180 day mortality compared with models only adjusting for inhospital factors (OR 1.25 (95% CI 1.11 to 1.41)).

**Table 2:** Differences in recognition and treatment of STEMIs between patients with COPD and non-COPD patients

Inhospital treatment and diagnosis	COPD N (%)	Non-COPD N (%)	Unadjusted OR (95% CI)	Minimally adjusted OR (95% CI)*	Adjusted OR (95% CI)†
Initial diagnosis other than definite STEMI (for final diagnosis is STEMI)	3080 (23.9)	24 752 (19.9)	1.26 (1.21 to 1.32)	1.28 (1.23 to 1.34)	1.24 (1.19 to 1.30)
Initial diagnosis other than ACS	1186 (9.2)	7398 (6.0)	1.59 (1.50 to 1.71)	1.68 (1.64 to 1.73)	1.52 (1.42 to 1.62)
Primary PCI	4108 (31.8)	44 177 (35.6)	0.84 (0.81 to 1.87)	0.69 (0.67 to 0.71)	0.87 (0.83 to 0.92)
Thrombolysis	5449 (42.6)	52 414 (42.7)	0.99 (0.96 to 1.03)	1.00 (0.96 to 1.03)	0.96 (0.91 to 1.10)

Time to reperfusion	COPD Minutes (median IQR)	Non-COPD Minutes (median IQR)	Unadjusted exponentiated regression coefficient (95% CI)	Minimally adjusted exponentiated regression coefficient (95% CI)*	Adjusted exponentiated regression coefficient (95% CI)†
Time to reperfusion from admission (overall)	37.1 (21.8–67.7)	35.0 (21.8–63.4)	1.07 (1.04 to 1.09)	1.05 (1.03 to 1.07)	1.04 (1.02 to 1.07)
Time to reperfusion from admission (initial diagnosis other than STEMI)	152.9 (74.3–705.6)	109.2 (50.2–260.0)	1.44 (1.24 to 1.67)	1.35 (1.16 to 1.58)	1.47 (1.15 to 1.88)
Time to reperfusion from admission (initial diagnosis STEMI)	35.0 (21.8–63.4)	35.0 (21.8–61.2)	1.04 (1.01 to 1.06)	1.03 (1.01 to 1.05)	1.03 (1.00 to 1.05)

Discharge treatment	COPD n (%)	Non-COPD n (%)	Unadjusted OR (95% CI)	Minimally adjusted OR* (95% CI)	Adjusted OR (95% CI)†
Discharge on β blockers	5776 (44.7)	94 784 (76.4)	0.25 (0.24 to 0.26)	0.25 (0.24 to 0.26)	0.26 (0.25 to 0.27)
Discharge on ACE inhibitor or angiotensin receptor blocker	9579 (74.2)	96 508 (77.8)	0.83 (0.79 to 0.86)	0.87 (0.83 to 0.91)	0.89 (0.85 to 0.93)
Discharge on aspirin	10 344 (80.1)	102 925 (82.9)	0.83 (0.79 to 0.87)	0.87 (0.83 to 0.92)	0.90 (0.85 to 0.94)
Discharge on statin	10 373 (80.4)	102 785 (82.8)	0.85 (0.81 to 0.89)	0.88 (0.84 to 0.93)	0.91 (0.86 to 0.95)
Discharge on thienopyridine	7799 (60.4)	77 543 (62.5)	0.91 (0.88 to 0.95)	0.96 (0.92 to 1.01)	0.98 (0.94 to 1.03)

*Adjusted for age, sex smoking status and calendar year.

†Adjusted for age, sex, smoking status, calendar year, drugs on admission and comorbidities.

ACS, acute coronary syndromes; COPD, chronic obstructive pulmonary disease; STEMI, ST-elevation myocardial infarction.

**Table 3:** Mortality after STEMI

	Adjusted for age, sex, smoking status and year	Adjusted for model 1 variables and comorbidities and drugs on arrival	Adjusted for models 1 and 2 variables and diagnostic delay	Adjusted for models 1 and 2 variables and use of reperfusion and time to reperfusion	Adjusted for models 1–4	Adjusted for models 1–4 variables and secondary prevention
	Model 1	Model 2	Model 3	Model 4	Model 5	Model 6
	OR (95% CI)					
Inhospital mortality	1.27 (1.16 to 1.39)	1.24 (1.10 to 1.41)	1.20 (1.06 to 1.36)	1.11 (0.94 to 1.31)	1.13 (0.99 to 1.29)	–
180-day mortality	1.43 (1.29 to 1.58)	1.45 (1.33 to 1.59)	1.43 (1.32 to 1.54)	1.46 (1.32 to 1.62)	1.45 (1.31 to 1.61)	1.25 (1.11 to 1.41)

All Odds ratios compare patients with COPD with non-COPD patients.

COPD, chronic obstructive pulmonary disease; STEMI, ST-elevation myocardial infarction.

After adjusting for inhospital processes, the estimated attributable risk of inhospital death following a STEMI due to COPD in patients with COPD decreased from 19.4% (95% CI 9.1% to 29.1%) to 11.5% (95% CI −1.0% to 22.4%). After adjusting for inhospital processes, the estimated attributable risk for death at 180 days due to COPD in patients with COPD following a STEMI decreased from 31.0% (95% CI 24.8% to 37.1%) to 20.0% (95% CI 9.9% to 29.1%).

### Non-ST-elevation myocardial infarctions

1. Describing the problem: differences in mortality after MI between patients with COPD and non-COPD patients
  After adjusting for age, sex, smoking status, calendar year, comorbidities and drugs on arrival, mortality in patients with COPD was higher than non-COPD patients in hospital (OR 1.34 (95% CI 1.24 to 1.45)) and 180 days after discharge (OR 1.56 (95% CI 1.47 to 1.65)).
2.  Possible inhospital mechanisms: differences in diagnosis and management after an MI between patients with COPD and non-COPD patients
  Results from the comparison of treatment and diagnosis after a non-STEMI are presented in table 4. Patients with COPD were more likely to have an initial diagnosis other than ACS after a non-STEMI (OR 1.46 (95% CI 1.41 to 1.50)). After a non-STEMI, patients with COPD were less likely to receive angiography in hospital (OR 0.69 (95% CI 0.66 to 0.71)). Patients with COPD were less likely to receive any of the secondary prevention drugs on discharge, apart from thienopyridines, compared with non-COPD patients, β blockers significantly more so than other secondary prevention drugs (OR 0.25 (95% CI 0.24 to 0.25)).
3.  Accounting for differences in mortality after MI between patients with COPD and non-COPD patients in terms of hospital processes

**Table 4:** Differences in recognition and inhospital treatment of non-STEMIs between patients with COPD and non-COPD patients

	COPD N (%)	Non-COPD N (%)	Unadjusted OR (95% CI)	Minimally adjusted OR* (95% CI)	Adjusted OR (95% CI)†
Inhospital treatment and diagnosis					
Initial diagnosis other than MI	9551 (45.2)	50 365 (35.5)	1.50 (1.46 to 1.54)	1.68 (1.64 to 1.73)	1.46 (1.41 to 1.50)
Angiography in hospital	8629 (40.9)	74 304 (52.2)	0.77 (0.76 to 0.79)	0.63 (0.61 to 0.65)	0.69 (0.66 to 0.71)
Discharge treatment					
Discharge on βblockers	6632 (31.4)	925 059 (64.9)	0.25 (0.24 to 0.26)	0.24 (0.23 to 0.25)	0.25 (0.24 to 0.25)
Discharge on ACE inhibitor or angiotensin receptor blocker	12 762 (60.4)	89 368 (63.0)	0.90 (0.87 to 0.92)	0.91 (0.88 to 0.94)	0.94 (0.91 to 0.97)
Discharge on aspirin	15 234 (72.1)	106 652 (75.1)	0.86 (0.83 to 0.88)	0.88 (0.85 to 0.91)	0.91 (0.88 to 0.94)
Discharge on statin	15 141 (71.7)	104 804 (73.8)	0.90 (0.87 to 0.93)	0.90 (0.87 to 0.93)	0.93 (0.90 to 0.96)
Discharge on thienopyridine	11 277 (53.4)	78 233 (55.1)	0.93 (0.90 to 0.96)	0.95 (0.91 to 0.98)	0.97 (0.94 to 1.01)

*Adjusted for age, sex smoking status and calendar year.†Adjusted for age, sex, smoking status, calendar year, drugs on admission and co-morbidities.

COPD, chronic obstructive pulmonary disease; MI, myocardial infarction; STEMI, ST-elevation myocardial infarction.

When compared with results found in (1), inhospital mortality was reduced after adjusting separately for both delay in diagnosis (OR 1.29 (95% CI 1.19 to 1.39)) and use of angiography (OR 1.18 (95% CI 1.09 to 1.29); table 5)). After adjusting for both delay in diagnosis and use of angiography the OR for inhospital mortality was 1.16 (95% CI 1.07 to 1.26). Inhospital factors also appeared to explain some of the mortality difference after a non-STEMI at 180 days. For mortality at 180 days, the OR was reduced from 1.56 (95% CI 1.47 to 1.65) to 1.37 (95% CI 1.31 to 1.44). Use of secondary prevention also seemed to explain some of the gap in mortality at 180 days. Compared with the model which only adjusted for inhospital processes, the OR for mortality at 180 days was reduced from 1.37 (95% CI 1.31 to 1.44) to 1.26 (95% CI 1.17 to 1.35).

**Table 5:** Mortality after non-STEMI. All ORs compare patients with COPD with non-COPD patients

	Adjusted for age, sex, smoking status and year	Adjusted for model 1 variables and comorbidities and drugs on arrival	Adjusted for models 1 and 2 variables and diagnostic delay	Adjusted for models 1 and 2 variables and use of angiography in hospital	Adjusted for models 1–4 variables	Adjusted for models 1–4 variables and secondary prevention
	Model 1	Model 2	Model 3	Model 4	Model 5	Model 6
	OR (95% CI)					
Inhospital mortality	1.40 (1.30 to 1.52)	1.34 (1.24 to 1.45)	1.29 (1.19 to 1.39)	1.18 (1.09 to 1.29)	1.16 (1.07 to 1.26)	–
180-day mortality	1.63 (1.56 to 1.70)	1.56 (1.47 to 1.65)	1.45 (1.38 to 1.52)	1.43 (1.34 to 1.50)	1.37 (1.31 to 1.44)	1.26 (1.17 to 1.35)

COPD, chronic obstructive pulmonary disease; STEMI, ST-elevation myocardial infarction.

After adjusting for inhospital processes, the estimated attributable risk for inhospital death following a non-STEMI due to COPD in patients with COPD decreased from 25.4% (95% CI 19.4% to 31.0%) to 13.8% (95% CI 6.5% to 21.6%). After adjusting for inhospital processes, the estimated attributable risk for death at 180-days due to COPD in patients with COPD following a non-STEMI decreased from 35.9% (95% CI 32.0% to 39.4%) to 20.6% (95% CI 14.5% to 25.9%).

## Discussion

### Summary of main findings

For STEMIs, some of the in inhospital mortality difference between patients with COPD and non-COPD patients may be attributable to delays in diagnosis and use of and increased time to reperfusion. Some of the increased mortality for STEMIs at longer time periods up to 6 months may be attributable to decreased use of secondary prevention medicines, especially β blockers, but not inhospital processes. For non-STEMIs, some of the difference in inhospital mortality may be attributable to delays in diagnosis and decreased use of angiography shortly after MI. Some of the increased mortality for non-STEMIs at longer time periods up to 6 months may be attributable to decreased use of secondary prevention medicines, and to inhospital delays in diagnosis and decreased use of angiography in hospital.

### Interpretation and comparison with other studies

Several studies have shown both the increased risk for death following MI for people with COPD and differences in management. These studies specifically showed reduced use of secondary prevention and pPCI after a STEMI in patients with COPD,^5^ ^10^ ^16–18^ these findings have been replicated here. This study has also shown that these differences in treatment are possible explanations for some of the mortality gap at the population level for both STEMIs and non-STEMIs. In particular, we were able to make use of the detailed timing variables available in MINAP to investigate differences in time to reperfusion after a STEMI.

For STEMIs, we found that diagnosis of MI is more likely to be delayed for patients with COPD compared with non-COPD patients, and that time to reperfusion is longer after a STEMI. We also showed that the effect of delay in diagnosis of MI on the time to reperfusion was greater in patients with COPD compared with non-COPD patients. Patients with COPD were more likely to have a delay in diagnosis and the effect of this delay in diagnosis in time to reperfusion was more severe for them than non-COPD patients. The reason for the delay in diagnosis of MI in patients with COPD may be because symptoms of MI in patients with COPD may be incorrectly attributed to their COPD rather than an MI.

We found that after a non-STEMI, patients with COPD were less likely to receive angiography in hospital than non-COPD patients, and this explained some of the excess inhospital and 180-day mortality. Use of angiography is driven by risk scoring, and patients at moderate and higher risk of death within 6 months should be offered angiography within 96 h of admission to hospital after a non-STEMI.^19^ It is unclear why, as a population, that although patients with COPD are at a higher risk of mortality they are less likely to receive angiography in hospital.

After both STEMIs and non-STEMIs, patients with COPD were less likely to be prescribed secondary prevention medicines than non-COPD patients. This may only have been to a clinically relevant degree for β blockers. It is known that patients with COPD are less likely to be prescribed β blockers after an MI, and that prescribing them improves survival.^11^ This study has demonstrated that the increased mortality associated with not prescribing secondary prevention medicines could explain some of the mortality gap up to 6 months at the population level.

We found that recognition of MI in patients with COPD was impaired compared with non-COPD patients. However, all patients included in this analysis were eventually diagnosed with MI. This suggests that patients with COPD may be at higher risk of having a completely missed MI. Indeed, recent work has suggested that as many as 1 in 12 patients admitted to hospital with an exacerbation of COPD meet the criteria for MI, and that this represents underdiagnosis of MI in patients with COPD.^20^ However, as troponin may also be increased during stable periods of COPD,^21^ there is also a potential for overdiagnosis of MI in people with COPD. Any future intervention which aims to increase recognition of MI in people with COPD should also investigate the potential effects of overdiagnosis.

### Strengths and limitations

The major strengths of this study were its size, representativeness and level of detail on inhospital management and outcomes. The study included over 300 000 people and used data collected from all hospitals in the UK which admit patients for ACS. As secondary prevention treatment is known to be different for patients with COPD compared with non-COPD patients, only using first MIs allowed us to assess the effect of COPD on mortality after an MI without bias due to differences in previous treatment. Another strength of this study was our ability to separate factors which could explain increased inhospital mortality from increased mortality following discharge. If patients with COPD were more likely to die in hospital, as we found, the reasons that they did not receive certain treatments may have been because they were more likely to die before they received these treatments compared with non-COPD patients. In order to avoid this bias, for mortality at 180 days, we only analysed data for those who had survived until at least discharge. This also allowed the potential contribution of secondary prevention to the mortality gap to be investigated.

One of the limitations of this study is potential misclassification of COPD status. The strategy used to identify may have misclassified asthmatic smokers as patients with COPD, and may have misclassified patients with COPD as non-COPD patients. However, the prevalence of COPD in our study is similar to that of previous work in similar settings.^5^ ^10^ ^16^ ^22^ The presence of asthmatics in our COPD group and patients with COPD in the non-COPD group is likely to have biased our findings towards the null. However, this would not change our findings. In addition, a sensitivity analysis, which compared mortality for asthmatic patients compared with non-asthmatic patients found that mortality was not increased in the asthmatic group (see online supplementary material). One of the limitations of using an audit database such as MINAP is the lack of available data which would not have been collected at hospital admission. Ideally, information on COPD severity and cause of death would have been collected. In addition, ideally information on socioeconomic status would have been available as this is a potential confounder for the relationship between COPD and mortality after MI. Future studies should investigate the relationship between COPD severity and explanations for the mortality gap in patients with COPD after MI and cause of death in patients with COPD following MI.

### Conclusions

Patients with COPD appear to receive poorer treatment after an MI compared with non-COPD patients. These differences in recognition and treatment of MI seem to explain some of the mortality gap between patients with COPD and non-COPD patients both inhospital and at 6 months postdischarge. Delayed diagnosis, timing and use of reperfusion of a STEMI, use of angiography after a non-STEMI and use of secondary prevention medicines are all potential explanations for the mortality gap after MI in people with COPD.

**Key messages**

**What is already known on this subject?**

- People with chronic obstructive pulmonary disease (COPD) have both a higher risk for myocardial infarction (MI) and poorer long-term outcomes following MI. Previous studies have also shown that patients with COPD are less likely to receive β blockers on discharge after an MI and are less likely to receive PCI after an ST-elevation myocardial infarction (STEMI). Findings for differences in inhospital mortality have been mixed, with some studies finding higher mortality for patients with COPD and some studies finding no difference. The heterogeneity in findings may be due to differences in treatment practices. The extent to which differences in treatment can explain differences in mortality at the population level, the ‘mortality gap’, is unclear.

**What might this study add?**

- This study aimed to determine whether differences in inhospital treatment and discharge between patients with and without COPD could explain all or some of the difference in mortality for both inhospital and at 180 days postdischarge at the population level. We found that delayed diagnosis of MI, decreased use of reperfusion and increased time to reperfusion after a STEMI, decreased use of angiography after a non-STEMI and decreased use of secondary prevention medicines might all explain some of the mortality gap for people with COPD after an MI.

**How might this impact on clinical practice?**

- We have found that differences in potentially modifiable inhospital processes may explain some of the mortality gap between patients with and without COPD after an MI. Clinicians need to be aware that it may be easier to miss MIs in people with COPD and may need to be aware of more unusual presentations of MI in people with COPD. In addition, our results suggest that patients with COPD may benefit from more aggressive treatment after an MI.

## Supplementary Material

Web appendix

Web figure
