# Supplementary material for: Closing the mortality gap after a myocardial infarction in people with and without chronic obstructive pulmonary disease
Source: Heart. 2015 Mar 12;101(14):1103–10. doi: 10.1136/heartjnl-2014-307251 (PMC4516011; doi:10.1136/heartjnl-2014-307251)
Supplement: Web appendix [file heartjnl-2014-307251-s1.pdf]

Supplementary appendix

Sensitivity analyses

As a sensitivity analysis, mortality after an MI was investigated comparing those who, for the purposes of this study, we presumed had asthma (those with a record of obstructive airway disease but no smoking history).

Additional analysis in optimal and sub-optimal care groups

We also compared mortality at 180 days for COPD patients and non-COPD patients within strata of optimal care, adjusted for age, sex, year, smoking status and co-morbidities. For STEMIs, patients were categorised as having optimal care if they had no delay in diagnosis, use of reperfusion and use of secondary prevention. For non-STEMIs, patients were categorised as having optimal care if they had no delay in diagnosis, use of angiography in-hospital, and use of secondary prevention. Sub-optimal care was defined as any factor missing from optimal care. We compared optimally treated COPD patients to optimally treated non-COPD patients; and non-optimally treated COPD patients to non-optimally treated non-COPD patients. We also compared mortality between those with optimal care and non-optimal care at 180 days among COPD patients.

Results

Difference in time to reperfusion between COPD patients and non-COPD patients among those without a delay in diagnosis

The difference in time to reperfusion between COPD and non-COPD patients was not apparent among patients who did not have a delay in diagnosis (median time to reperfusion 35.0 minutes (IQR, 21.8-63.4) for COPD patients, and 35.0 minutes (IQR, 21.8-61.2) for non-COPD patients).

Adjusted analysis also showed no difference in time to reperfusion for COPD patients compared to non-COPD patients among those who did not have a delay in diagnosis (ratio of geometric means 1.03, 95% CI 1.00 to 1.05).

Sensitivity analysis with asthmatic patients

When in-hospital mortality after an MI was investigated for people who we presume to have asthma were compared to non-asthmatics, no difference in mortality was found in analysis adjusted for age, sex, smoking status, calendar year, co-morbidities and drugs on arrival (OR 1.05, 95% CI 0.89-1.24 for STEMIs; OR 1.05, 95% CI 0.91-1.22 for non-STEMIs).

Additional analysis in optimal and sub-optimal care groups

After a STEMI, the effect of COPD on mortality at 180 days in the non-optimal care group (OR 1.39, 1.29-1.51; non-optimally treated COPD patients compared to non-optimally treated non-COPD patients) was comparable to that in the optimal care group (OR 1.44, 1.08-1.94; optimally treated COPD patients compared to optimally treated non-COPD patients). After a non-STEMI, the effect of COPD on mortality at 180 days in the non-optimal care group (OR 1.53, 1.45-1.61; non-optimally treated COPD patients compared to non-optimally treated non-COPD patients) was lower than that in the optimal care group (OR 1.80, 1.36-2.37; optimally treated COPD patients compared to optimally treated non-COPD patients). Among COPD patients, having optimal treatment was associated with lower risk of death at 180 days after both a STEMI (OR 0.31, 0.23-0.42; optimally treated COPD patients compared to non-optimally treated COPD patients) and a non-STEMI (OR 0.34, 0.26-.43; optimally treated COPD patients compared to non-optimally treated COPD patients).
