# Supplementary figures and images for: Closing the mortality gap after a myocardial infarction in people with and without chronic obstructive pulmonary disease
Source: Heart. 2015 Mar 12;101(14):1103–10. doi: 10.1136/heartjnl-2014-307251 (PMC4516011; doi:10.1136/heartjnl-2014-307251)

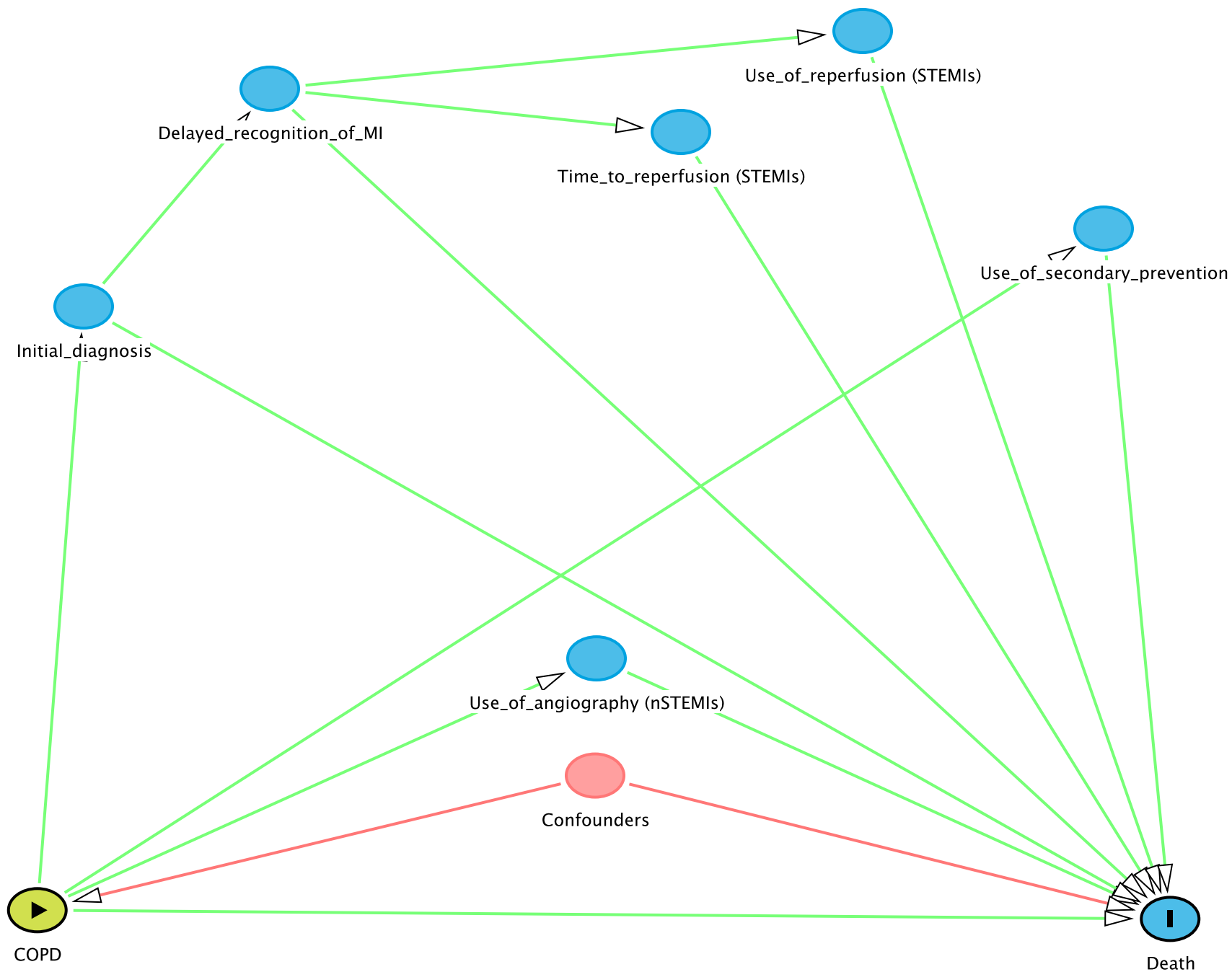

Supplement: Web figure [file heartjnl-2014-307251-s2.pdf]
